# Photodynamic therapy with paclitaxel-encapsulated indocyanine green-modified liposomes for breast cancer

**DOI:** 10.3389/fonc.2024.1365305

**Published:** 2024-03-07

**Authors:** Mariko Ishizuka, Masaki Kaibori, Fusao Sumiyama, Yoshiharu Okamoto, Akiko Suganami, Yutaka Tamura, Kengo Yoshii, Tomoharu Sugie, Mitsugu Sekimoto

**Affiliations:** ^1^Department of Surgery, Kansai Medical University, Osaka, Japan; ^2^WOLVES HAND Advanced Veterinary Medical Institute, Osaka, Japan; ^3^Department of Bioinformatics, Chiba University, Chiba, Japan; ^4^Department of Mathematics, Kyoto Prefectural University of Medicine, Kyoto, Japan

**Keywords:** photodynamic therapy, breast cancer, indocyanine green, immunogenic cell death, drug delivery system

## Abstract

**Background:**

Photodynamic therapy (PDT) involves the administration of a photosensitizing agent and irradiation of light at an excitation wavelength that damages tumor cells without causing significant damage to normal tissue. We developed indocyanine green (ICG)-modified liposomes in which paclitaxel (PTX) was encapsulated (ICG-Lipo-PTX). ICG-Lipo-PTX accumulates specifically in tumors due to the characteristics of the liposomes. The thermal and photodynamic effects of ICG and the local release of PTX by irradiation are expected to induce not only antitumor effects but also cancer immunity. In this study, we investigated the antitumor effects of ICG-Lipo-PTX in breast cancer.

**Methods:**

The antitumor effects of ICG-Lipo-PTX were examined in xenograft model mice subcutaneously implanted with KPL-1 human breast cancer cells. ICG-Lipo-PTX, ICG-Lipo, or saline was administered intraperitoneally, and the fluorescence intensity was measured with a fluorescence imaging system (IVIS). Intratumor temperature, tumor volume, and necrotic area of tumor tissue were also compared. Next, we investigated the induction of cancer immunity in an allogeneic transplantation model in which BALB-MC mouse breast cancer cells were transplanted subcutaneously in the bilateral inguinal region. ICG-Lipo-PTX was administered intraperitoneally, and PDT was performed on only one side. The fluorescence intensity measured by IVIS and the bilateral tumor volumes were compared. Cytokine secretory capacity was also evaluated by ELISPOT assay using splenocytes.

**Results:**

In the xenograft model, the fluorescence intensity and temperature during PDT were significantly higher with ICG-Lipo-PTX and ICG-Lipo in tumor areas than in nontumor areas. The fluorescence intensity in the tumor area was reduced to the same level as that in the nonirradiated area after two times of irradiation. Tumor growth was significantly reduced and the percentage of necrotic area in the tumor was higher after PDT in the ICG-Lipo-PTX group than in the other groups. In the allograft model, tumor growth on day 14 in the ICG-Lipo-PTX group was significantly suppressed not only on the PDT side but also on the non-PDT side. In addition, the secretion of interferon-γ and interleukin-2 was enhanced, whereas that of interleukin-10 was suppressed, in the ICG-Lipo-PTX group.

**Conclusion:**

The PDT therapy with ICG-Lipo-PTX may be an effective treatment for breast cancer.

## 1 Introduction

Breast cancer is the most common malignant disease in women, and its incidence and mortality rates are increasing worldwide (1, 2). About 75% of breast cancers are hormone receptor positive, and endocrine therapy has been used alone or in combination with surgical treatment, chemotherapy, molecular targeted therapy, and radiation therapy. The combination of endocrine therapy with CDK4/6 inhibitors significantly prolongs progression-free survival for hormone receptor-positive, human epidermal growth factor type 2-negative metastatic or recurrent breast cancer (3, 4). However, hormone receptor-positive breast cancer patients still face many challenges, including acquisition of treatment resistance and late recurrence. The combination of a taxane and an anthracycline is widely used as an adjuvant or neoadjuvant chemotherapy for breast cancer. Paclitaxel (PTX) is a taxane and has long been used to treat many cancers. The main mechanism of action of PTX involves its binding to microtubules, thereby contributing to microtubule stabilization and inhibiting cell division. In addition to its superior antitumor effects, PTX has been shown to reactivate the immune response against cancer by acting on tumor-associated macrophages (5). However, there are several side effects, including nausea and peripheral neuropathy, and it is also difficult to use PTX to safely treat patients with deteriorating health.

Photodynamic therapy (PDT) is a minimally invasive therapy that is used to selectively treat cancerous lesions with low energy, using a photochemical reaction between a photosensitive substance that accumulates in the tumor and laser light irradiation. In contrast to chemotherapy and irradiation therapy, which induce apoptosis via cell cycle checkpoint and p53 activation due to DNA damage, PDT induces an acute stress response, including mitochondrial damage and cytochrome c release (6). PDT does not cause cumulative toxicity and can be administered repeatedly to the same tumor if it responds (7). Photoimmunotherapy is an approach that combines PDT and immunotherapy to induce an antitumor immune response by inducing cytotoxic T cells (CTLs) to target neoantigens released from cancer tissue (8).

**Figure 1:**
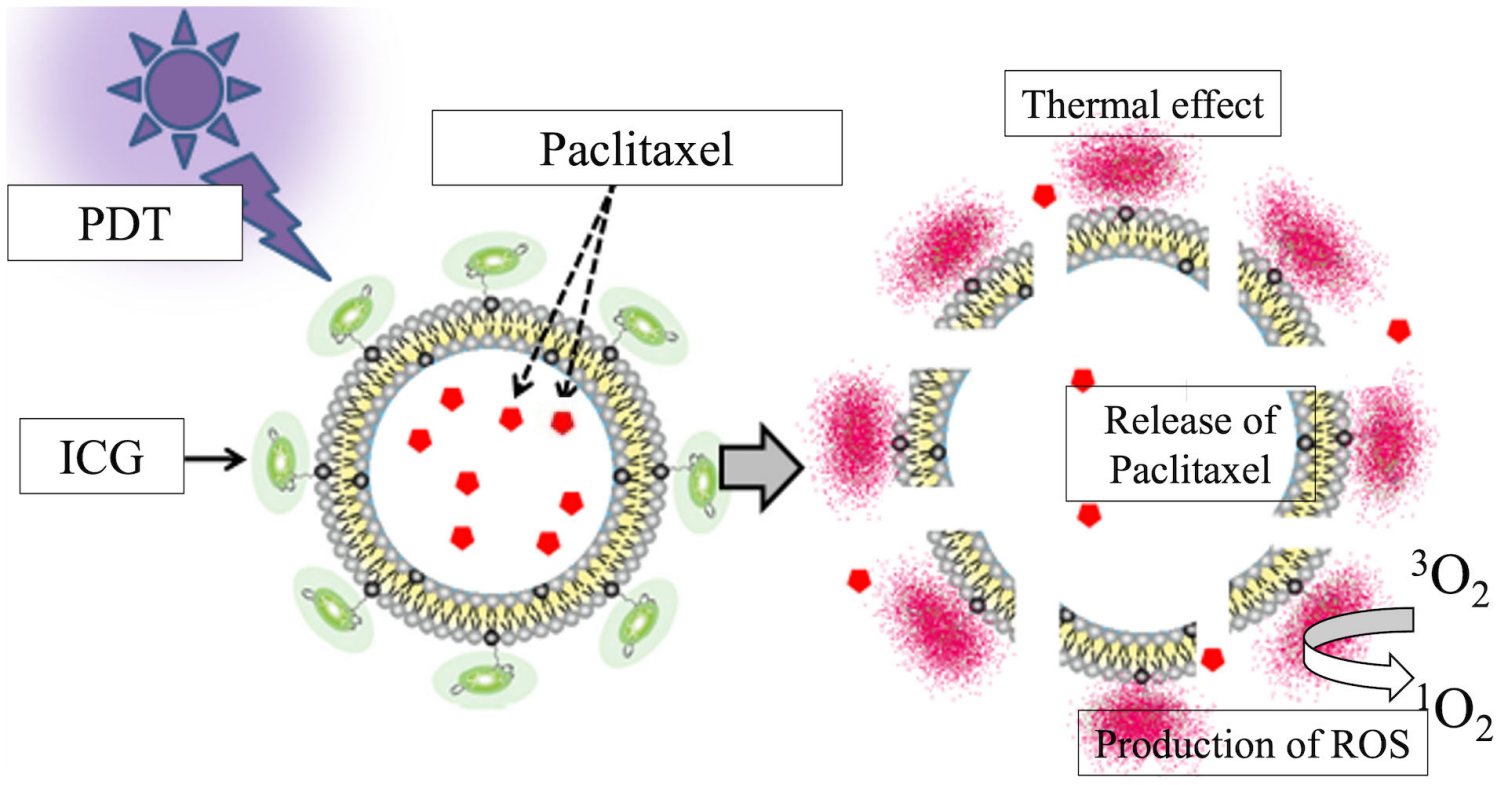
Schematic of the ICG-Lipo-PTX preparation. Indocyanine green (ICG) is bound to the surface of 200-nm-diameter liposomes via their C18 chains. Near-infrared irradiation at 810 nm produces heat and singlet oxygen species due to the thermal and photodynamic effects of ICG. The structural change in ICG causes disruption of the lipid bilayer of the liposome and local release of the encapsulated paclitaxel.

Near-infrared photoimmunotherapy (NIR-PIT) is a recently developed targeted molecular cancer therapy that combines a target-specific photosensitizer based on the infrared phthalocyanine dye IR700 with a monoclonal antibody against epidermal growth factor receptor (EGFR) (9). NIR-PIT has been shown to activate a systemic immune response against the target tumor through induction of immunogenic cell death (ICD) and to exert antitumor effects not only in primary tumors but also in distant metastases (10, 11). NIR-PIT is also known to further enhance the permeability and retention of tumor blood vessels (the enhanced permeability and retention [EPR] effect), a phenomenon called super-enhanced permeable retention (SUPR) (12, 13). Indocyanine green (ICG) reacts to near-infrared rays of around 800 nm in wavelength to generate heat (14, 15) and reacts to near-infrared rays of 600–800 nm to generate singlet oxygen (16, 17). ICG-modified liposomes (ICG-Lipo), in which the photosensitizing agent ICG is modified on the surface of liposomes with a particle size that accumulates tumor-specifically via the EPR effect, may represent a novel type of photoimmunotherapy. Based on these properties, it was shown that irradiation of ICG with near-infrared light at approximately 800 nm produces thermal and photodynamic effects that inhibit tumor growth more effectively compared with hyperthermia alone (18). The antitumor effect of ICG-Lipo against spontaneous tumors in animals has already been reported (19, 20). ICG-Lipo can also be used as a carrier for multiple drugs (21). We have developed ICG-Lipo-PTX, in which PTX is encapsulated inside ICG-Lipo (**Figure 1**). In addition to the aforementioned thermal and photodynamic effects of ICG-Lipo-PTX, the structural change in ICG disrupts the lipid bilayer of liposomes and releases the encapsulated PTX into the tumor region.

In this study, we investigated the antitumor effects of ICG-Lipo-PTX in human breast cancer. We also evaluated whether ICG-Lipo-PTX can induce cancer immunity using an allogeneic transplantation model.

## 2 Materials and methods

### 2.1 Cell lines

KPL-1 human breast cancer cells, which were established from the carcinomatous effusion of a postmenopausal patient with breast cancer who had become resistant to hormone therapy (22), were purchased from Kawasaki Medical School (Okayama, Japan) and maintained in Dulbecco’s modified Eagle’s medium (Wako, Osaka, Japan) supplemented with 10% fetal bovine serum (BioWest, Logan, UT, USA) and 1% penicillin-streptomycin solution (Cosmo Bio Company, Tokyo, Japan). BALB-MC mouse breast cancer cells, which were established from a spontaneous mammary tumor in a 17-month-old female BALB/c mouse (23), were purchased from the Japanese Collection of Research BioResources (Osaka, Japan) and maintained in Eagle’s minimum essential medium (Wako) supplemented with 10% fetal bovine serum (BioWest) and 1% penicillin-streptomycin solution (Cosmo Bio Company).

### 2.2 Animals

Four-week-old female BALB/c nude mice or BALB/c mice were purchased from Oriental Bio Service (Kyoto, Japan) and used in experiments at 5 weeks of age. The mice were housed at 22°C under a 12-h/12-h light/dark cycle with free access to food and water. All animal care and experiments were performed according to ARRIVE and PREPARE guidelines (24, 25) and were approved by the Ethics Committee for Animal Experiments of Kansai Medical University (approval no. 20-111, 21-012).

### 2.3 Preparation of ICG-Lipo and ICG-Lipo-PTX

ICG-Lipo and ICG-Lipo-PTX were prepared in four synthetic stages. (i) ICG derivatives, serving as a near-infrared fluorescent probe (ICG-C18), were prepared as described previously (26). (ii) ICG-C18 was mixed with 1,2-dioleoyl-sn-glycero-3-phosphocholine and 1,2-distearoyl-sn-glycero-3-phosphoethanolamine-N- [azido(polyethylene glycol)-5000] and then dissolved in a mixed organic solvent of CH_3_OH/CHCl_3_ (volume ratio: 1/9). A thin lipid film was formed by removing the solvent under reduced pressure. (iii) An aqueous buffered solution (phosphate-buffered saline) was added to a thin lipid film for ICG-Lipo dispersion, and phosphate-buffered saline with PTX (0.4 mg/mL) was added for ICG-Lipo-PTX dispersion. (iv) To confirm the standard for the EPR effect, the liposome dispersions were filtered 11 times through a 0.1-mm pore polycarbonate filter attached to the LiposoFast-Stabilizer (Avestin Inc., Ottawa, Canada) at room temperature.

### 2.4 Animal models

In the human breast cancer model, KPL-1 cells (5 × 10^6^) were transplanted under the right dorsal skin of BALB/c nude mice. When tumors reached 5–7 mm in diameter, animals were randomly assigned to one of the following six groups: ICG-Lipo-PTX group with or without PDT (ICG-Lipo-PTX+PDT, ICG-Lipo-PTX), ICG-Lipo group with or without PDT (ICG-Lipo-PTX+PDT, ICG-Lipo-PTX), or saline group with or without PDT (PDT-only, control). For each group, 100 μL ICG-Lipo (20 mg/mL ICG) was administered intraperitoneally on the first day of treatment.

In the mouse breast cancer model, BALB-MC cells (5 × 10^6^) were transplanted subcutaneously in the bilateral inguinal region of BALB/c mice. When tumors reached 5–7 mm in diameter, animals were randomly assigned to two groups: ICG-Lipo-PTX+PDT group and PDT-only group. Animals in the ICG-Lipo-PTX group were administered 100 µL ICG-Lipo-PTX intraperitoneally on the first and seventh days of treatment. Animals in the PDT-only group were administered 100 µL saline intraperitoneally.

In animal experiments, the observation period after drug administration was 14–21 days, and sufficient care was taken not to cause pain during operations, including imaging using the IVIS equipment and drug administration by inhalation or intraperitoneal injection of a triad of anesthetics. If the maximum diameter of the tumor exceeded 20 mm (27, 28), or if the animal lost 25% or more of body weight during the 7-day period, the animal was euthanized using anesthetics.

### 2.5 Fluorescence imaging and PDT

After 100 μL ICG-Lipo-PTX containing 0.2 mg/mL ICG was administered intraperitoneally to model mice anesthetized with isoflurane, the fluorescence intensity of ICG was compared between tumor and nontumor (contralateral subcutaneous) areas using an IVIS system (PerkinElmer, Waltham, MA, USA) to confirm drug localization. The fluorescence intensity was measured using 780-nm excitation light and an 845-nm filter. Fluorescence imaging was also performed with IVIS as needed for 14 days after administration to examine changes in localization with PDT and time course.

The protocol for PDT in the human breast cancer model was as follows. At 48 h after administration of saline, ICG-Lipo, or ICG-Lipo-PTX, model mice were anesthetized with a triad of anesthetics (0.3 mg/kg medetomidine, 4.0 mg/kg midazolam, and 5.0 mg/kg butorphanol) and irradiated with an LED light source (ASAHI SPECTRA, Tokyo, Japan). Irradiation was performed at a wavelength of 810 nm at a distance of 1 cm from the tumor (the fluence rate was 100 mW/cm^2^, and the irradiation time was 10 min). Three days after irradiation, a second irradiation was performed under the same conditions.

For PDT in the mouse breast cancer model, the following protocol was used. Model mice were administered saline or ICG-Lipo-PTX, and PDT was performed under the same conditions as described above from days 3 to 7. For tumors in the bilateral inguinal region, two courses of five irradiations on days 3–7 were performed on the left side of the tumor, and no light irradiation was performed on the right side. Tumor volume was then measured up to 14 days after treatment. The tumor volume was calculated using the following formula: (long axis × short axis^2^)/2.

### 2.6 Measurement of the temperature inside the tumor

Intratumor temperature was measured using a thermologger (AM800; ANRITSU METER Co., Tokyo, Japan). A 20-gauge needle was inserted into the tumor, and the measuring terminal was inserted into the tumor through the lumen of the needle.

### 2.7 Histological examination

In the human breast cancer model, on day 14 of treatment, subcutaneous tumors were removed from 4–5 mice in each group, fixed in formalin, embedded in paraffin, and sectioned using standard protocols. Sections were stained with hematoxylin-eosin (HE), and the necrotic area was measured. The necrotic area was defined as the sum of areas showing nuclear enrichment, anucleation, cytoplasmic cleavage, and eosinophilia and was expressed as a percentage of the total tumor area. Measurements were performed using a BX-43 microscope (Olympus, Tokyo, Japan) and a cell calculator MODEL F410N (ERMA, Tokyo, Japan).

In the mouse breast cancer model, tumor tissue was removed on day 14 of treatment, fixed in formalin, and embedded in paraffin using standard protocols to prepare sections for immunostaining. Immunohistochemistry was performed using rat anti-CD4 antibodies or rat anti-CD8 antibodies (BD Pharmingen, San Diego, CA, USA). The number of CD4- or CD8-positive cells per square millimeter was counted in 10 randomly selected fields of view, excluding obvious necrotic areas, and the average was used.

### 2.8 Enzyme-linked immunospot assay

In the mouse breast cancer model, ICG-Lipo-PTX or saline was administered intraperitoneally, and PDT was performed only on the left-sided tumor according to the PDT protocol described above. After 14 days of treatment, mice were euthanized, and splenocytes were collected. ELISPOT assays were then used to detect interferon (IFN)-γ, interleukin (IL)-4, IL-2, and IL-10 in order to determine whether coculture with BALB-MC cells caused cytokine secretion to vary between the ICG-Lipo-PTX group and PDT-only group. Splenocytes (6.0 × 10^5^) and BALB-MC cells (6.0 × 10^5^) were added to the plates, and the plates were placed in an incubator containing 5% CO_2_ for 24 h at 37°C. An Immunospot S6 Analyzer (Cellular Technology Limited, Cleveland, OH, USA) was used to automatically count the number of spots.

### 2.9 Statistical analysis

Data are expressed as means ± standard errors (SEs). The tumor necrosis area was analyzed by one-way analysis of variance followed by multiple comparisons using the Tukey-Kramer test. Fluorescence intensity and ELISPOT assay results were analyzed using the Wilcoxon rank sum test, and tumor volume trends were analyzed using Steel’s multiple comparison test. Results with *P* values less than 0.05 were considered statistically significant.

## 3 Results

### 3.1 Fluorescence imaging in the human breast cancer model

**Figure 2:**
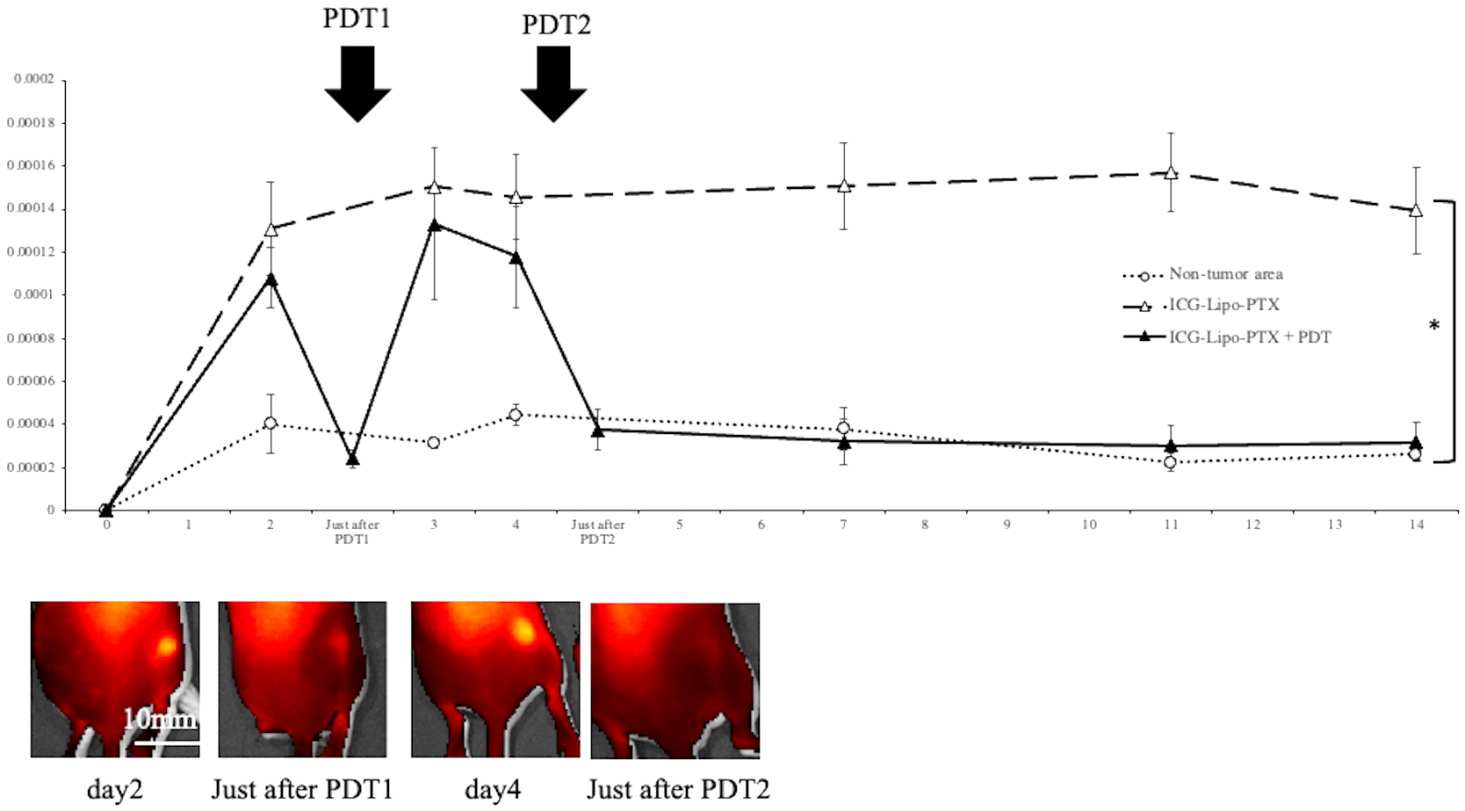
Fluorescence imaging in mouse models of subcutaneous tumors. Fluorescence intensity trends in subcutaneous tumors in the ICG-Lipo-PTX+PDT and ICG-Lipo-PTX groups and IVIS imaging of the tumors in the ICG-Lipo-PTX+PDT group are shown. Fluorescence imaging was performed using an IVIS system in ICG-Lipo-PTX treated mice. After administration ICG-Lipo-PTX, the brightness of the tumor was measured at the indicated times (ICG-Lipo-PTX+PDT: ▲, ICG-Lipo-PTX: △, non-tumor areas: ○). On days 2 and 4 of administration, PDT (810 nm, 10 min) was performed in the ICG-Lipo-PTX group. *P < 0.05 between the tumor and nontumor areas. Data are presented as means ± SEs (n = 5 mice/group).

The fluorescence intensity in ICG-Lipo-PTX-treated mice was measured by IVIS, and images of the tumor area before and after PDT are shown (**Figure 2**). The images revealed strong fluorescence in the tumors derived from tumor cells transplanted under the right dorsal skin in the mice on day 2, and the first PDT course resulted in a tendency for fluorescence weakening in the tumor area. Then, strong fluorescence was again observed in the tumor area on day 4; however, the fluorescence almost disappeared after the second PDT course. Forty-eight hours after ICG-Lipo-PTX administration, the fluorescence intensity in the tumor area was significantly higher than that in the nontumor area (contralateral back, *P* < 0.05). In the ICG-Lipo-PTX+PDT group, the fluorescence intensity of the tumor area quickly decreased immediately after irradiation but then increased again; after the second irradiation on day 4, the fluorescence intensity decreased again and remained low thereafter. By contrast, in the ICG-Lipo-PTX group, the fluorescence intensity of the tumor remained significantly higher than that of the nontumor area during the 14-day observation period. The fluorescence intensity in the ICG-Lipo group was also evaluated using the IVIS system and was similar to that in the ICG-Lipo-PTX group (data not shown).

### 3.2 Temperature trends inside the tumor

**Figure 3:**
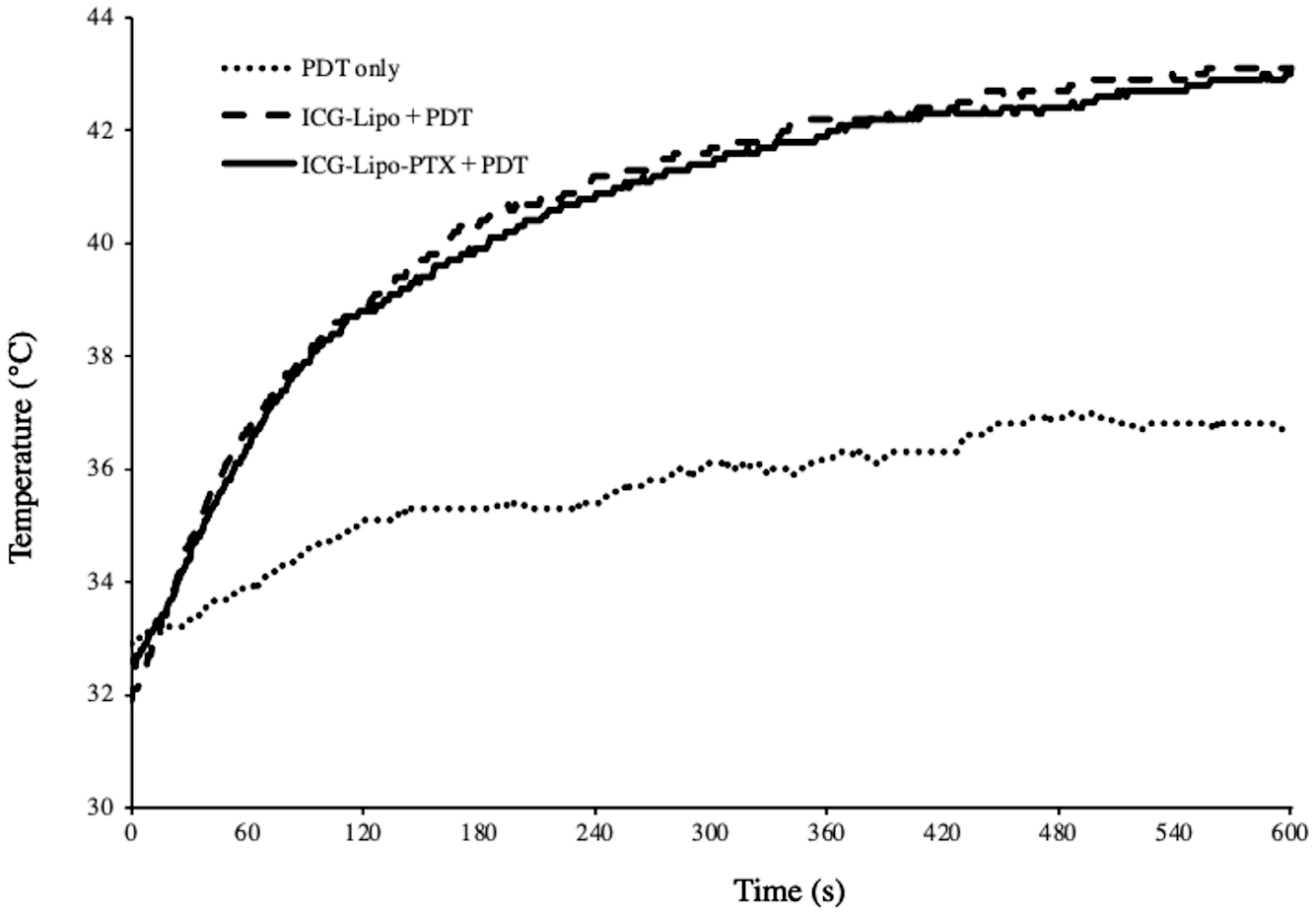
Temperature trends inside the tumor after PDT. Two days after administration of ICG-Lipo, PDT (810 nm, 10 min) was performed with PDT-only (dotted line), ICG-Lipo+PDT (dashed line), or ICG-Lipo-PTX+PDT (solid line). The temperature trends inside the tumor were measured continuously during the 10-min PDT. Three independent experiments were performed, and representative results are shown.

The intratumor temperature during 10 min of PDT was measured using a thermologger (**Figure 3**). After the start of irradiation, the temperature in tumors from the ICG-Lipo group rose quickly, exceeding 40°C in about 3 min. The temperature continued to rise and reached 42°C in about 5 min after the start of irradiation, which was expected to induce a thermal effect. During the 10-min irradiation, the maximum intratumor temperatures were 43.0°C in the ICG-Lipo-PTX+PDT group and 43.1°C in the ICG-Lipo+PDT group; however, no burns or other side effects were observed on the skin at the irradiated area.

### 3.3 Antitumor effects of ICG-Lipo-PTX in human breast cancer

**Figure 4:**
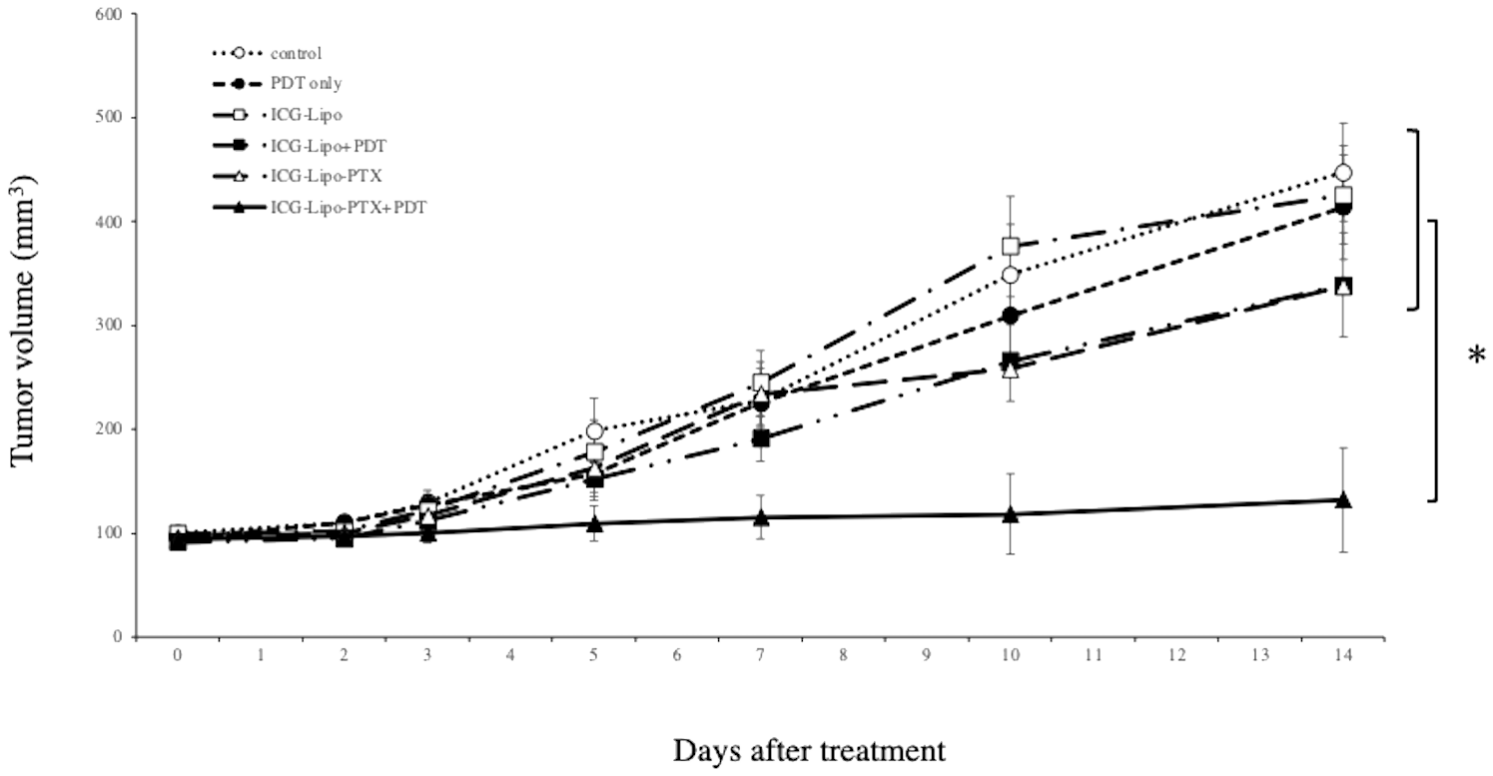
Antitumor effects of ICG-Lipo in the subcutaneous tumor model inoculated with KPL-1 cells. In this model, 5 × 10^6^ KPL-1 cells were transplanted subcutaneously into BALB/c nude mice. Mice were randomly assigned to six groups: no treatment (○), PDT-only (●), ICG-Lipo (□), ICG-Lipo+PDT (■), ICG-Lipo-PTX (△), and ICG-Lipo-PTX+PDT (▲). Tumor volume trends were observed from the day of ICG-Lipo administration to day 14. PDT was performed 2 and 4 days after ICG-Lipo administration. Data are expressed as means ± SEs (n = 8 mice/group). *P < 0.005 between the ICG-Lipo-PTX+PDT and other groups.

The antitumor effects of ICG-Lipo-PTX in KPL-1 human breast cancer cells were examined in nude mice. BALB/c nude mice transplanted with KPL-1 cells subcutaneously in the back were assigned to six groups (n = 8 per group): ICG-Lipo-PTX+PDT, ICG-Lipo-PTX, ICG-Lipo+PDT, ICG-Lipo, PDT-only, and control. Tumor volume was then evaluated (**Figure 4**). PDT was performed twice in each group. On day 14 of treatment, the tumor volume (± SE) in the ICG-Lipo-PTX+PDT group was 132.4 ± 9.7 mm^3^, which was significantly smaller than those in the other groups (ICG-Lipo-PTX: 337.7 ± 31.2 mm^3^, ICG-Lipo+PDT: 338.7 ± 27.9 mm^3^, ICG-Lipo: 425.5 ± 36.8 mm^3^, PDT-only: 413.9 ± 50.2 mm^3^, control: 447.4 ± 47.1 mm^3^; *P* < 0.05). No adverse events, such as weight loss, were observed in any of the six groups of mice (**Supplementary Figure 1**).

### 3.4 Histological examination

**Figure 5:**
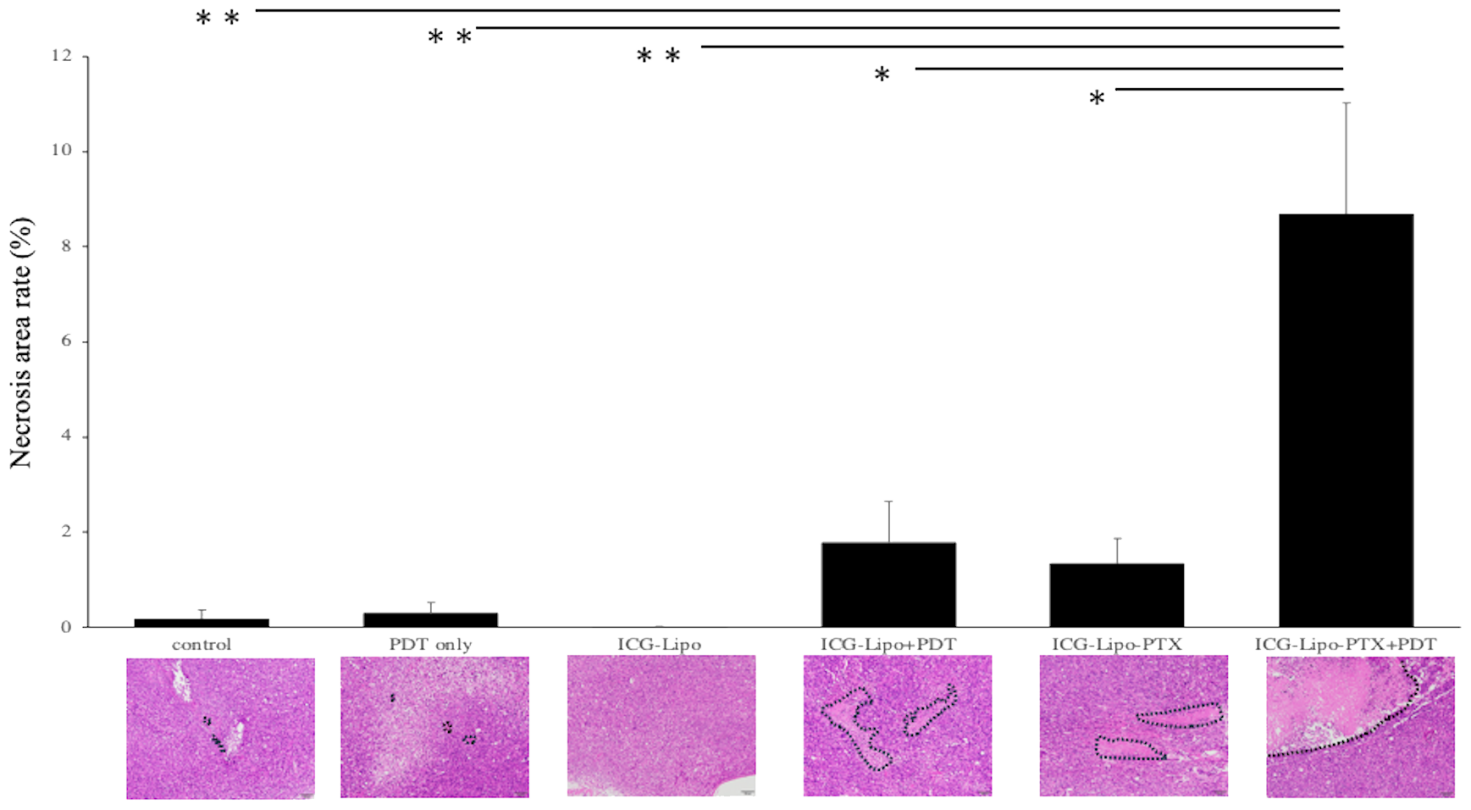
Pathological examination of subcutaneous tumors. On day 14 of ICG-Lipo administration, subcutaneous tumors in mice were excised and stained with HE. The percentage of necrotic area within the tumor, along with HE-stained images, are shown. Data are expressed as means ± SE (n = 4–5 mice/group). The necrotic areas are indicated by dashed lines in the HE-stained images. **P < 0.01 between the ICG-Lipo-PTX+PDT group and the ICG-Lipo, PDT-only, and control groups; *P < 0.05 between the ICG-Lipo-PTX+PDT group and the ICG-Lipo-PTX and ICG-Lipo.

Subcutaneous tumors in the six groups (n = 4 in each group) were removed on day 14 of treatment, and the necrotic areas were evaluated by HE staining. The average of the percentage of necrotic area in the tumors of each group and a representative HE-stained image of the six groups are shown. (**Figure 5**). In the Lipo-PTX+PDT group, the necrotic area encircled by the dashed line accounted for 8.69 ± 2.34% of the total tumor tissue; this was significantly greater than those of the other groups (ICG-Lipo-PTX: 1.35% ± 0.51%, *P* < 0.05; ICG-Lipo+PDT: 1.78% ± 0.86%, *P* < 0.05; ICG-Lipo: 0.01% ± 0.01%, *P* < 0.001; PDT-only: 0.31% ± 0.21%, *P* < 0.001; control: 0.19% ± 0.18%, *P* < 0.001).

### 3.5 Fluorescence imaging in the mouse breast cancer model

**Figure 6:**
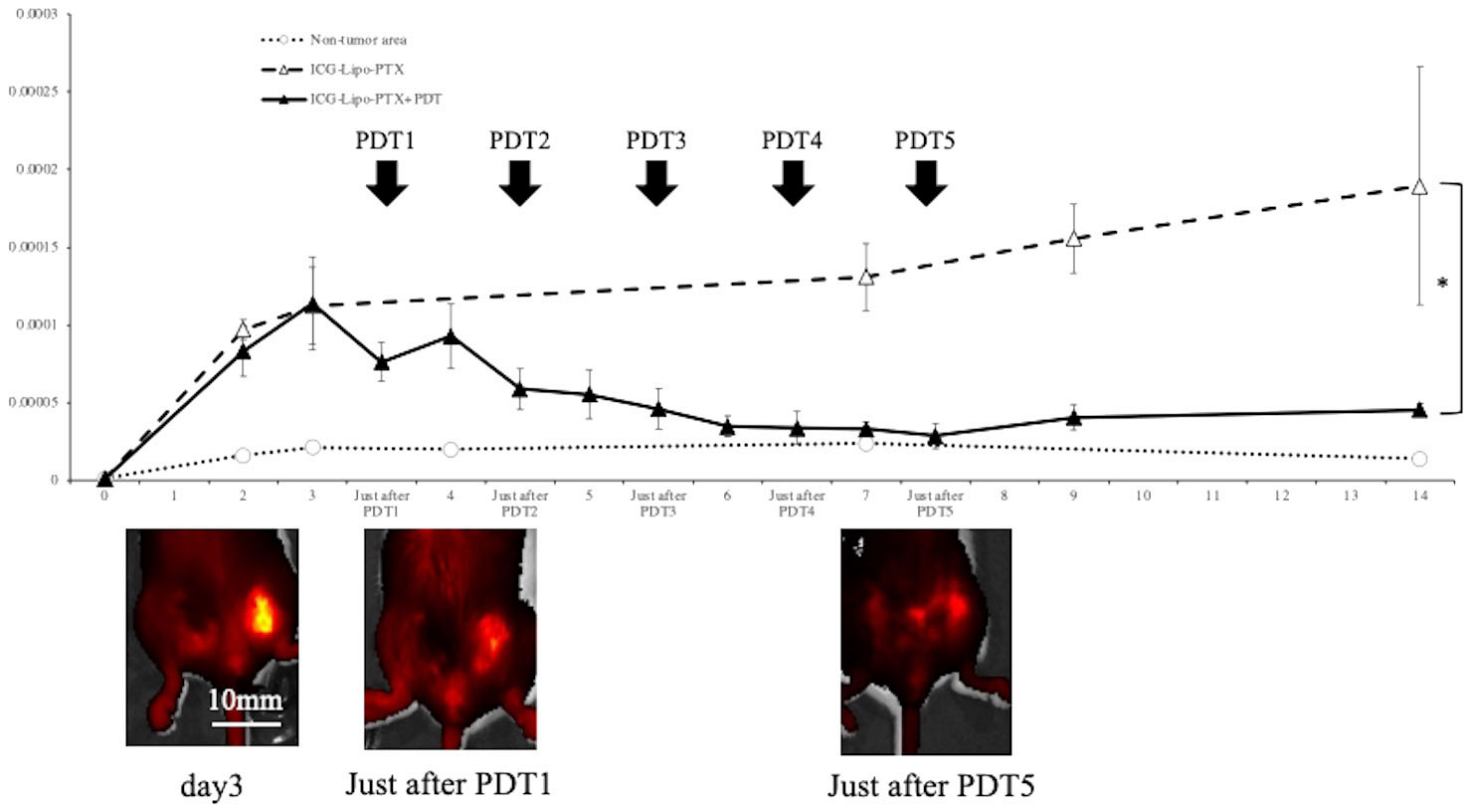
Fluorescence imaging in BALB/c mouse models of subcutaneous tumors. Fluorescence intensity trends in subcutaneous tumors in the ICG-Lipo-PTX+PDT and ICG-Lipo-PTX groups and IVIS imaging of tumors in the ICG-Lipo-PTX+PDT group are shown. (ICG-Lipo-PTX+PDT: ▲, ICG-Lipo-PTX: △, nontumor areas: ○). After the first irradiation, re-accumulation was observed in the tumor area. However, after the second irradiation (PDT2), no recovery of fluorescence intensity was observed.

To examine the pharmacokinetics of ICG-Lipo-PTX in mouse breast cancer tumor tissue, the fluorescence intensity trends of the ICG-Lipo-PTX group were measured by IVIS, similar to the approach in the human breast cancer model (**Figure 6**). Images taken by IVIS showed that the fluorescence intensity of ICG-Lipo-PTX +PDT group mice showed a progressive decrease in fluorescence accumulated in the tumor in the left inguinal region with each successive PDT. As in the human breast cancer model, tumor-specific accumulation of ICG-Lipo-PTX and a decrease in accumulation due to irradiation were observed. Although re-accumulation was observed in the tumor area after the first PDT, there was no re-accumulation after the second irradiation, and the fluorescence intensity in the tumor area decreased with the number of irradiations. Five irradiations were required for the fluorescence intensity in the tumor area to equal that in the nontumor area. The fluorescence intensity in the nonirradiated group remained high during the observation period up to day 14.

### 3.6 Antitumor effects of ICG-Lipo-PTX in mouse breast cancer

**Figure 7:**
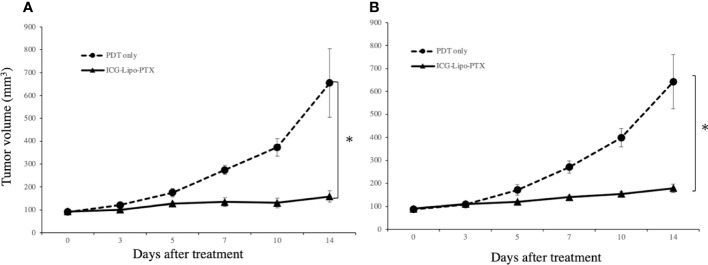
Antitumor effects of ICG-Lipo in the bilateral subcutaneous tumor model. In this model, 5 × 10^6^ BALB-MC cells were transplanted subcutaneously in the bilateral inguinal region of BALB/c mice. Tumor volume was observed from the day of the first administration of ICG-Lipo until day 14. PDT was performed only on tumors in the left inguinal region on days 3–5 after the day of ICG-Lipo administration (days 0 and 7). The tumor volume was evaluated on the **(A)** left side treated with PDT and **(B)** right side treated with no irradiation. Data are expressed as means ± SEs (n = 8 mice/group). *P < 0.005 between the ICG-Lipo-PTX and PDT-only groups.

In the mouse model in which BALB-MC cells were transplanted into the bilateral inguinal region, ICG-Lipo-PTX or saline was administered, and two courses of five irradiations on days 3–7 were then performed on the left side only. On day 14, the tumor volumes on the left side treated with PDT were 158.6 ± 24.2 mm^3^ in the ICG-Lipo-PTX group and 809.9 ± 149.9 mm^3^ in the PDT-only group (P < 0.001; **Figure 7A**). The tumor volumes on the right side on day 14 were 178.9 ± 18.3 mm^3^ in the ICG-Lipo-PTX group and 642.4 ± 118.5 mm^3^ in the PDT-only group (P < 0.001; **Figure 7B**). Tumor growth was significantly suppressed in the ICG-Lipo-PTX group on both sides.

### 3.7 T-cell immune responses in antitumor immunity

**Figure 8:**
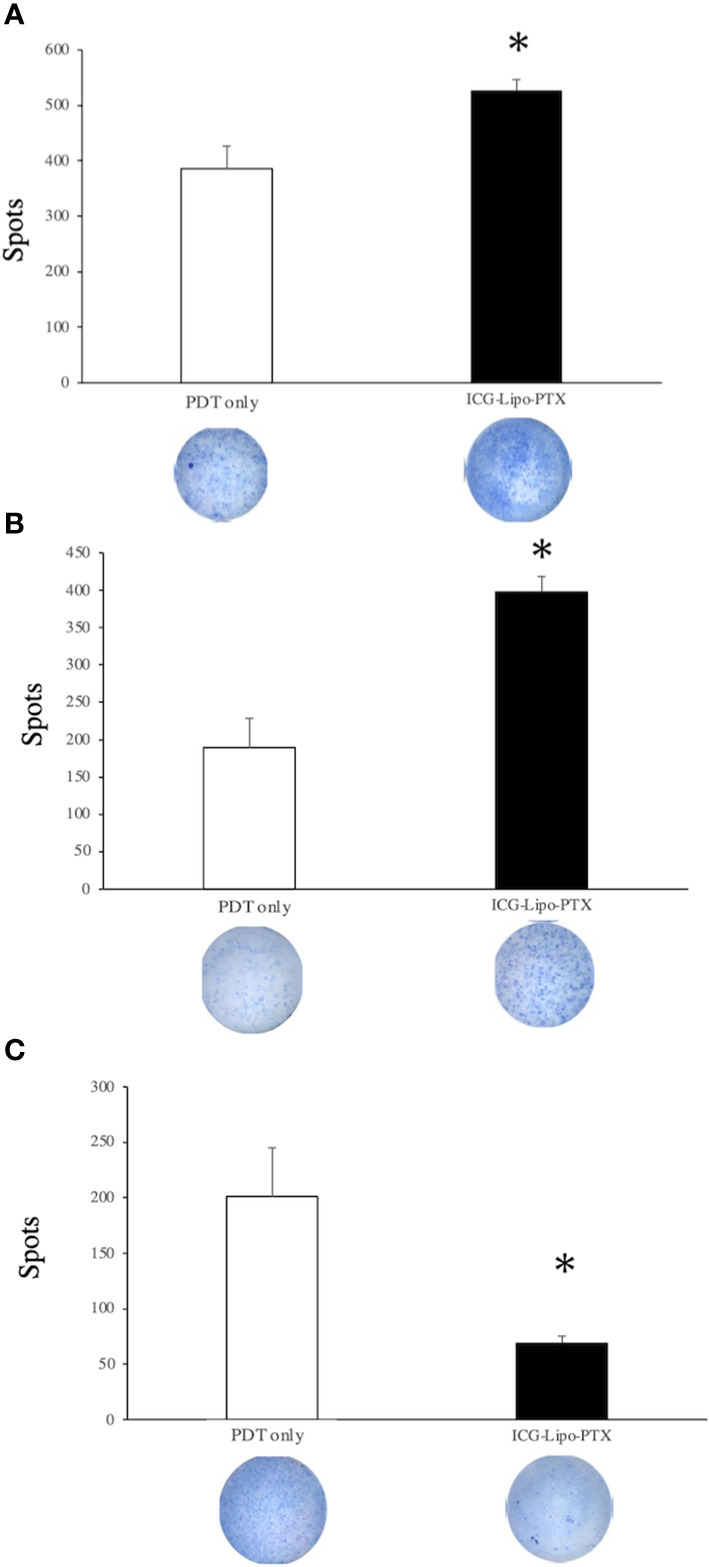
Changes in IFN-γ, IL-2, and IL-10 levels in the splenocytes in the treatment group. BALB/c mice transplanted with 5 × 10^6^ BALB-MC cells in the bilateral inguinal region were treated with PDT only or ICG-Lipo-PTX+PDT. PDT was performed on days 3–7. The second ICG-Lipo-PTX administration was on day 7, and PDT was performed on days 10–14. On day 14, ELISPOT assays for **(A)** IFN-γ, **(B)** IL-2, and **(C)** IL-10 were performed with splenocytes from each group. Data are expressed as means ± SEs (n = 6 mice/group). *P < 0.05 between the ICG-Lipo-PTX group and the PDT-only group.

In the mouse breast cancer model, splenocytes were isolated from mouse spleens, and the secretory levels of cytokines were compared between the ICG-Lipo-PTX and PDT-only groups (**Figure 8**). On day 14, the ICG-Lipo-PTX group showed significantly higher levels of IFN-γ and IL-2 than the PDT-only group, and IL-10 secretion was significantly suppressed in the ICG-Lipo-PTX group (n = 6; P < 0.05). Although IL-4 secretion levels were enhanced in the ICG-Lipo-PTX group, they were not significantly different from those in the PDT-only group (data not shown).

In further experiments, we evaluated the numbers of immune cells in tumor tissues in the ICG-Lipo-PTX group using immunohistochemistry. Bilateral subcutaneous tumor tissues were removed on day 14 of treatment, and immunohistochemistry for CD-4 and CD-8 was performed to compare the number of positive cells per unit area between the ICG-Lipo-PTX and PDT-only groups on the PDT and non-PDT sides (n = 4 per group). The number of CD-4- and CD-8 positive cells was increased in the ICG-Lipo-PTX group but was not significantly different from that in the PDT-only group (**Supplementary Figure 2**).

## 4 Discussion

In this study, we demonstrated that ICG-Lipo-PTX accumulated in a tumor-specific manner and that PDT to the tumor site produced an antitumor effect. Although the fluorescence intensity was decreased by PDT, re-accumulation was observed at 24–48 h after irradiation, and the intensity was greater than the peak of the first accumulation (**Figure 2**). We suggest that the first irradiation damaged the tumor cells and enhanced the local inflammatory response and that systemic circulating ICG-Lipo-PTX accumulated again in the tumor area due to the SUPR effect. Thus, ICG-Lipo-PTX had the thermal and photodynamic effects of ICG as well as the EPR effects of liposomes to deliver the encapsulated drug locally to the tumor. In the allograft model, however, the SUPR effect was not observed (**Figure 6**). We also examined the antitumor effect of two times of irradiations per ICG-Lipo-PTX administration in the allograft model. The results showed that the fluorescence intensity in the tumor area did not decrease to the same degree as that in the non-tumor area after only two times of irradiations, and the tumor suppressive effect was not significantly different between the groups (data not shown). In the allograft model, the dose of ICG-Lipo-PTX used in this study was rather low, and it is possible that the first irradiation did not induce enough damage; thus, more frequent irradiation was required to obtain an antitumor effect. Nanomicellar PTX effectively reaches the tumor and shows antitumor effects with as little as 20% of the normal dose, and no systemic toxicity has been reported, even after an overdose (29). ICG-Lipo provides an excellent drug delivery system and has the potential to provide antitumor effects with the minimum amount of drug required. Previously, we succeeded in reducing the amount of drug contained in ICG-Lipo to about 10% of the normal dose in companion animals (21). To reduce side effects and to take advantage of the fact that PDT does not cause cumulative toxicity, it is important to minimize the amount of drug in ICG-Lipo. A characteristic side effect of PTX is peripheral neuropathy, which increases in frequency in a volume-dependent manner (30). We suggest that reductions in the PTX dose and total dose through the efficient drug delivery system of ICG-Lipo-PTX are essential for the management of side effects and the completion of chemotherapy in the future clinical trials.

Cancer cell death pathways induced by PDT include apoptosis (31, 32), necroptosis (31, 33), and ferroptosis (34, 35). The main mechanism of PDT-induced cancer cell death is apoptosis stimulated by intracellular production of reactive oxygen species (32). Notably, the light dose of PDT can induce both apoptosis and necrosis (36). PDT itself has also been reported to induce ICD (11, 37). ICD is distinct from noninflammatory cell death mechanisms, including the elimination of senescent cells; the release of danger-associated molecular patterns (DAMPs) from dying tumor cells undergoing specific treatment, which results in immune cell infiltration and sustained local inflammation (38); and the release of DAMPs following ICD, which stimulates antigen-presenting cells and activates tumor-specific cytotoxic T cells (38–41). However, in this study, we found increases in the necrotic area (**Figure 5**) and in IFN-γ secretion (**Figure 8**) in the treated group. ICD is also known to increase IFN-γ secretion, reflecting T-cell activation (37). A tumor-suppressive effect equivalent to that observed on the left side treated with PDT was also observed on the right side (**Figure 6**), suggesting that DAMPs were released after cancer cell death on the PDT side, and that T-cell activation occurred, as reflected by increased IFN-γ secretion. As a result of the systemic induction of cancer immunity, tumor tissue on the non-PDT side may be attacked by cytotoxic T cells due to the induction of cancer immunity, resulting in an antitumor effect.

There were some limitations to this study. For example, there was no direct proof of induction of cancer immunity. There were more tumor-infiltrating CD4- and CD8-positive cells in the ICG-Lipo-PTX group on the PDT and non-PDT sides than in the control group, although the difference was not significant (**Supplementary Figure 2**). Some studies have reported that compared with NIR-PIT alone, more CD8-positive cells infiltrate tumors when the therapy is combined with anti-programmed death-1 antibodies, IL-15, and CTLA4 (42–44). Thus, PDT alone may inhibit the induction of immune cells into tumors owing to its tumor vascular-blocking effect (45). In addition to PTX, ICG-Lipo may potently induce immune cells to become tumors via their encapsulation of immune checkpoint inhibitors and other drugs. Furthermore, a recent study in the field of photoimmunotherapy described the development of an approach in which NIR-PIT targeted EGFR and human EGFR2 expressed in tumor tissues as well as cytotoxic T cells and regulatory T cells (9). Moreover, an approach in which multiple drugs with different mechanisms of action were encapsulated inside ICG-Lipo was shown to produce more potent antitumor effects (21). In the future, it may be possible to develop ICG-Lipo to simultaneously encapsulate multiple anticancer drugs, antihormonal drugs, and molecular-targeted drugs. Light irradiation can be used easily in breast cancer because of the proximity of the tumor to the body surface, and unlike radiotherapy, PDT itself has no cumulative toxicity; therefore, it can be safely performed for treatment of local recurrence or chest wall recurrence.

In conclusion, we demonstrated the antitumor effects of ICG-Lipo-PTX in human and mouse breast cancer cells. Necrotic cell death was observed in post-treatment tumor tissues, accompanied by increased secretion of IFN-γ and IL-2. We speculated that antitumor effects were obtained not only on the PDT side but also on the non-PDT side by induction of systemic cancer immunity.

## Data availability statement

The raw data supporting the conclusions of this article will be made available by the authors, without undue reservation.

## Ethics statement

Ethical approval was not required for the studies on humans in accordance with the local legislation and institutional requirements because only commercially available established cell lines were used. The animal study was approved by Ethics Committee for Animal Experiments of Kansai Medical University Tomoyuki Nakamura. The study was conducted in accordance with the local legislation and institutional requirements.

## Author contributions

MI: Writing – original draft. MK: Writing – original draft, Writing – review & editing. FS: Project administration, Writing – review & editing. YO: Methodology, Project administration, Resources, Writing – review & editing. AS: Methodology, Project administration, Resources, Writing – review & editing. YT: Methodology, Project administration, Resources, Writing – review & editing. KY: Formal analysis, Writing – review & editing. TS: Writing – review & editing. MS: Writing – review & editing.
