# Supplementary material for: Photodynamic therapy with paclitaxel-encapsulated indocyanine green-modified liposomes for breast cancer
Source: Front Oncol. 2024 Mar 7;14:1365305. doi: 10.3389/fonc.2024.1365305 (PMC10955121; doi:10.3389/fonc.2024.1365305)
Supplement: Supplementary file 1 [file DataSheet_1.docx]

Supplementary Material

Photodynamic therapy with paclitaxel-encapsulated indocyanine green-modified liposomes for breast cancer

Mariko Ishizuka^1^, Masaki Kaibori^1^, Fusao Sumiyama^1^, Yoshiharu Okamoto^2^, Akiko Suganami^3^, Yutaka Tamura^3^, Kengo Yoshii^4^, Tomoharu Sugie^1^, Mitsugu Sekimoto^1^

*** Correspondence:** Masaki Kaibori: kaibori@hirakata.kmu.ac.jp

## Supplementary Figures

**Supplementary Figure 1.** Body weights of mice in each group.

In this model, 5 × 10^6^ KPL-1 cells were transplanted subcutaneously into BALB/c mice. Body weights were compared between the beginning of treatment and at day 14 of treatment. Data are expressed as means ± SEs (n = 4–6 mice/group).

## Supplementary Figures

1. (B)

(C)　　　　　　　　　　　　　　　　　 (D)

BALB/c mice transplanted with 5 × 10^6^ BALB-MC cells in the bilateral inguinal region were treated with saline or ICG-Lipo-PTX. PDT was performed on days 3–7. The second ICG-Lipo-PTX administration was on day 7, and PDT was performed on days 10–14. On day 14 of treatment, bilateral subcutaneous tumors were harvested for (A) anti-CD4 immunostaining of the PDT side, (B) anti-CD4 immunostaining of the non-PDT side, (C) anti-CD8 immunostaining of the PDT side, and (D) anti-CD8 immunostaining of the non-PDT side. Although there was a trend toward higher numbers of immunostaining-positive cells in the ICG-Lipo-PTX group for both the PDT side and the non-PDT side, there were no statistically significant differences between this group and the PDT-only group (*P* = 0.2, 0.2, 0.34, and 0.47, respectively). The graphs present the numbers of CD8+ or CD4+ cells/mm^2^. Data represent means ± SEs (n = 4 mice/group).
